# Common SNP in hsa-miR-196a-2 increases hsa-miR-196a-5p expression and predisposes to idiopathic male infertility in Chinese Han population

**DOI:** 10.1038/srep19825

**Published:** 2016-01-25

**Authors:** Jing Lu, Hao Gu, Qiuqin Tang, Wei Wu, Beilei Yuan, Dan Guo, Yongyue Wei, Hong Sun, Yankai Xia, Hongjuan Ding, Lingqing Hu, Daozhen Chen, Jiahao Sha, Xinru Wang

**Affiliations:** 1 State Key Laboratory of Reproductive Medicine, Department of Reproduction, Nanjing Maternal and Child Health Care Hospital Affiliated to Nanjing Medical University, Nanjing 210004, China; 2 State Key Laboratory of Reproductive Medicine, Institute of Toxicology, Nanjing Medical University, Nanjing 210029, China; 3 Key Laboratory of Modern Toxicology of Ministry of Education, School of Public Health, Nanjing Medical University, Nanjing 210029, China; 4 State Key Laboratory of Reproductive Medicine, Department of Obstetrics, Nanjing Maternal and Child Health Care Hospital Affiliated to Nanjing Medical University, Nanjing 210004, China; 5 Wuxi Maternal and Child Health Hospital Affiliated to Nanjing Medical University, Wuxi 214002, China; 6 Department of Epidemiology and Biostatistics, Key Laboratory of Modern Toxicology of Ministry of Education, School of Public Health, Nanjing Medical University, Nanjing, 210029, China; 7 Department of Microbial and Molecular Systems, KULeuven, Kasteelpark Arenberg 20, B-3001 Leuven, Belgium; 8 State Key Laboratory of Reproductive Medicine, Nanjing Medical University, Nanjing 210029, China

## Abstract

MicroRNA plays an important role in spermatogenesis. Whether pre-miRNAs polymorphisms are associated with idiopathic male infertility remains obscure. In this study, 1378 idiopathic infertile males and 486 fertile controls were included between 2006 and 2014. Genotype of three polymorphisms (*hsa-mir-146a* rs2910164, *hsa-mir-196a-2* rs11614913, and *hsa-mir-499* rs3746444) and expression of miRNA in seminal plasma were examined by TaqMan method. The role of hsa-miR-196a-5p in cell proliferation, apoptosis and cell cycle were also examined in GC-2 cells. Our results demonstrated that rs11614913 of *hsa-miR-196a-2* was significantly associated with idiopathic infertility (TT vs. CT: *P* = 0.014; TT vs. CC: *P* = 0.005; TT vs. CT + CC: *P* = 0.003). In following stratified analysis, we found that rs11614913 exhibited a significantly higher risk of asthenospermia, oligozoospermia and azoospermia. However, no significant association was observed between the other two polymorphisms and idiopathic male infertility risk. In a genotype-expression correlation analysis, rs11614913 CC was significantly associated with elevated expression of hsa-miR-196a-5p (*P* < 0.05). Additionally, apoptosis levels were significantly increased in hsa-miR-196a-5p mimic treated GC-2 cells, while decreased in hsa-miR-196a-5p inhibitor treated GC-2 cells. Our data revealed a significant relationship between *hsa-miR-196a-2* polymorphism and idiopathic male infertility.

Infertility affects about one in six couples attempting pregnancy, with the man being responsible in approximately half of the cases^1^. Despite a significant improvement in the diagnostic work-up of infertile men, the cause of abnormal spermatogenesis in about half of all cases remains unknown^2^. A significant proportion of male infertility is accompanied by abnormal semen quality including oligozoospermia and asthenospermia, which is generally assumed to be the result of genetic alterations^3^,^4^. In the last 50 years, sperm density has declined approximately 1.5% every year in the USA and 3% in Europe^5^. However, the underlying molecular and genetic mechanisms for spermatogenesis and maturation remained unclear.

MicroRNAs (miRNAs) are small single stranded regulatory RNAs that are produced through a multistep process generating a primary transcript-miRNA (pri-miRNA) and a precursor-miRNA (pre-miRNA). It has been suggested that miRNAs are involved in various biological process, including cell proliferation, cell death, stress resistance, and fat metabolism^6^,^7^,^8^. Allelic variants in the sequences of mature miRNAs, as well as in those of pri- and pre-miRNAs, represent a particularly interesting potential source of phenotypic diversity of genetic diseases and they may contribute directly to disease susceptibility^9^.

As a specific miRNA has the potential of regulating the expression of hundreds of target mRNAs, single nucleotide polymorphisms (SNPs) in miRNAs may produce more significant functional consequences and represent an ideal candidate for disease prediction. At present, global alterations of miRNAs are often found in cancers^10^. For example, Hu *et al*. performed a screening for common SNPs in miRNA sequence and have identified four SNPs (rs2910164, rs2292832, rs11614913, and rs3746444) located at the pre-miRNA regions of *hsa-mir-146a*, *hsa-mir-149*, *hsa-mir-196a-2*, and *hsa-mir-499*, respectively^11^. They observed the association of the rs11614913: T > C variant genotype with a significantly increased risk of non-small cell lung cancer. A number of case-control studies were consequently performed in breast cancer^12^, hepatocellular carcinoma^13^, head and neck cancer^14^, and gastric cancer^15^. On the other hand, the rs2910164 within the *hsa-mir-146a* was associated with papillary thyroid carcinoma^16^ and hepatocellular carcinoma^17^. Moreover, the rs11614913 in the *hsa-mir-196a-2* and the rs3746444 in the *hsa-mir-499* were both associated with the risk of suffering breast cancer^18^. Because very few SNPs in the SNP databases (the NCBI dbSNP database, build 127; http://www.ncbi.nlm.nih.gov/projects/SNP/) may be incorrect and not applicable for the population-based studies in Chinese populations, we surveyed common (i.e., minor allele frequency > 0.05) SNPs located in pre-miRNAs and their surrounding regions. We then selected three most commonly studied pre-miRNA SNPs (*hsa-mir-146a* rs2910164 C > G, *hsa-mir-196a-2* rs11614913 T > C and *hsa-mir-499* rs3746444 A > G) and evaluated their associations with the susceptibility of male infertility in our present study.

In many species the hsa-mir-196a-2 appears to be expressed from intergenic regions in HOX gene clusters. Yekta *et al*.^19^ reported that miR-196 are expressed from *HOX* gene clusters in mammals, and that *HOX* genes in these clusters are targets of miR-196. HOX clusters are groups of related transcription factor genes crucial for numerous developmental programs in animals. miR-146a is primarily involved in the regulation of inflammation and other process that function in the innate immune system. miR-499 is associated with the regulation of embryonic stemness, cell proliferation, cell size and apoptosis^20^. Recently, several studies have showed that miRNAs are associated with spermatogenesis and sperm maturation^21^,^22^. Impaired sperm quality may be factors underlying infertility and possibly predisposing to cancer diseases^23^. Previous studies have reported that infertile males may have an increased risk of subsequently developing cancer^24^,^25^, such as prostate cancer. Therefore, in addition to cancer, we have reason to believe that functional alteration of miRNAs caused by SNPs may also contribute to the pathogenesis of idiopathic male infertility. However, relevant epidemiological studies are still very limited. So, it is urgent to understand SNPs in reproduction as possible action. In this study, we hypothesized that these functional SNPs were associated with idiopathic male infertility. To validated this hypothesis, we performed genotyping analyses for rs11614913 T > C and the other two common SNPs (rs2910164 G > C, and rs3746444 A > G) located at pre-miRNA regions and evaluated their association with the susceptibility of idiopathic male infertility in a case-control study of 1378 infertility cases and 486 fertile controls in a Han Chinese population.

## Results

### The relevant characteristics of the subjects

The final population consisted of 1864 Han Chinese, composed of 486 fertile controls and 1378 infertile males. Among the 1378 infertile males, 927 were normozoospermia, 405 were asthenospermia, 131 were oligozoospermia and 140 were azoospermia. The frequency distributions of selected characteristics of the cases and controls are presented in Table 1.

### Associations between polymorphisms and spermatogenic impairment

The associations of the three SNPs with normospermia, asthenospermia, oligozoospermia and azoospermia are presented in Table 2. The observed genotype frequencies for these three polymorphisms were all in agreement with that expected under the Hardy-Weinberg equilibrium in the controls, except for rs2910164 (*P* < 0.010). In Table 2, 95% CI was adjusted for the age, BMI, smoking and alcohol status in cases and controls because of their potential confounding effects.

Initially, we overviewed the frequency distribution between the total case group and the control group, genotypes of rs2910164 and rs3746444 showed no significant differences. However, rs11614913 was significantly associated with idiopathic infertility (TT vs. CT: OR = 1.34; 95% CI: 1.06–1.69; *P* = 0.014; TT vs. CC: OR = 1.53; 95% CI: 1.13–2.06; *P* = 0.005; TT vs. CT + CC: OR = 1.39; 95% CI: 1.12–1.73; *P* = 0.003; respectively).

In order to further assess the possibility of an association between the three variants and a particular aspect of semen parameters, we subdivided the case group into four subgroups on the basis of sperm concentration and sperm motility: normozoospermia, asthenospermia, oligozoospermia and azoospermia. Since semen parameters showed large variation, we presented them in median (range). Sperm concentration and motility in all infertile patients were 33.34 (6.45–76.28) × 10^6^/ml and 32.60 (7.74–52.27) %, respectively. Sperm concentration and motility in the normospermia group were 70.30 (40.67–113.71) × 10^6^/ml and 53.79 (43.38–66.09) %, respectively. In the asthenospermia group, sperm motility was 7.33 (0–21.62) %. In the oligozoospermia group, sperm concentration was 6.45 (3.14–10.86) × 10^6^/ml. Sperm motility in azoospermia group was 0. As presented in Table 2, no significant differences of distribution frequencies were identified for the variants of rs2910164, rs3746444, among the control group and each case group. Similar results were found for the frequency of rs11614913 between the control group and the normospermia group, while we found significant association between rs11614913 and other subgroups of idiopathic male infertility.

For rs11614913, the TT genotype was significantly lower in cases of asthenospermia group, oligozoospermia group and azoospermia group when compared with the control group. Therefore, logistic regression analysis revealed that compared with TT genotype, subjects in asthenospermia group carrying the heterozygous CT and CC genotype had a significantly increased risk of idiopathic male infertility (OR = 1.78, 95% CI: 1.31–2.43, *P* < 0.001 and OR = 1.99, 95% CI: 1.36–2.91, *P* < 0.001). When we combined the rs11614913 CT and CC genotype, assuming a co-dominant allele effect, the combined CT + CC variant genotypes were strongly associated with a risk of idiopathic male infertility (OR = 1.84, 95% CI: 1.37–2.46, *P* < 0.001). For oligozoospermia group, individuals with at least one C allele were at a significantly increased risk of idiopathic male infertility compared with those harboring TT genotype (TT vs. CT: OR = 1.93, 95% CI: 1.16–3.21, *P* = 0.011; TT vs. CC: OR = 3.87, 95% CI: 2.23–6.72, *P* < 0.001; TT vs. CT + CC: OR = 2.48, 95% CI: 1.55–3.97, *P* < 0.001; respectively). The same trend was found regarding the azoospermia group (TT vs. CT: OR = 1.60, 95% CI: 1.01–2.51, *P* = 0.043; TT vs. CC: OR = 2.20, 95% CI: 1.29–3.76, *P* = 0.005; TT vs. CT + CC: OR = 1.76, 95% CI: 1.15–2.70, *P* = 0.009; respectively). These result suggests that the C allele of rs11614913 (T > C) may contribute to the risk of idiopathic male infertility.

The sensitivity analyses using re-sampling statistical methods retained consistent results (Additional file 1 for bootstrap and permutation analyses; Additional file 2 for jackknife analysis), which showed the robustness of the findings.

### Seminal plasma expression level of hsa-miR-196a-5p in idiopathic infertile males and fertile controls

**Figure 1:**
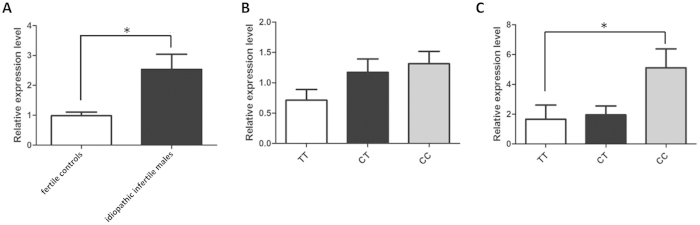
Expression level of hsa-miR-196a-5p in seminal plasma of idiopathic infertile males and fertile controls with different genotypes of rs11614913. (**A**) Expression level of hsa-miR-196a-5p in idiopathic infertile males and fertile controls (n = 80 for fertile controls; n = 107 for idiopathic infertile males). (**B**) Expression level of hsa-miR-196a-5p in fertile controls with different genotypes (n = 37 for TT genotype; n = 29 for CT genotype; n = 14 for CC genotype). (**C**) Expression level of hsa-miR-196a-5p in idiopathic infertile males with different genotypes (n = 35 for TT genotype; n = 49 for CT genotype; n = 25 for CC genotype). Data are shown as mean ± SEM in each group. Statistical analyses for hsa-miR-196a-5p expression level were performed by Mann-Whitney test. Significant difference is marked with *P < 0.05.

In order to investigate the association between genotypes of rs11614913 and mature miRNA expression level of hsa-miR-196a-5p, we conducted a correlation analysis between rs11614913 genotypes and the expression level of hsa-miR-196a-5p. Physical map of rs11614913 in *hsa-mir-196a-2* gene and mature miRNA hsa-miR-196a-5p in the genomic is shown in Additional file 3. Seminal plasma samples were available for 107 idiopathic infertile males and 80 fertile controls with different genotypes in the present study. We found that the expression level of hsa-miR-196a-5p was significantly increased in idiopathic infertile males compared with fertile controls (Fig. 1). The expression of hsa-miR-196a-5p in males with CC genotype was significantly higher than that of TT genotype in idiopathic infertile males. However, no significant association was found between CT and TT genotypes in idiopathic infertile males and between different genotypes in fertile controls (Fig. 1).

### Functional effects of hsa-miR-196a-5p on proliferation, cell cycle progression, and apoptosis in GC-2std (GC-2) cells

**Figure 2:**
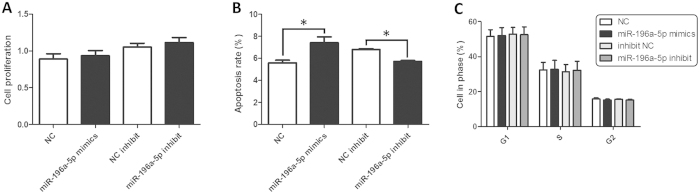
The role of hsa-miR-196a-5p in cell proliferation, apoptosis and cycle cycle *in vitro*. (**A**) The role of hsa-miR-196a-5p in cell proliferation. An MTT cell viability assay was performed at 24 h after the transfection of GC-2 cells with equal concentrations of hsa-miR-196a-5p mimics and hsa-miR-196a-5p inhibitor. (**B**) The role of hsa-miR-196a-5p in cell apoptosis. (**C**) The role of hsa-miR-196a-5p in cell cycle. For comparison, the expression levels of hsa-miR-196a-5p mimics or hsa-miR-196a-5p inhibitor transfected cells were compared with their respective negative controls (*P < 0.05).

Furthermore, we analysis the impact of hsa-miR-196a-5p on cells proliferation, apoptosis and cell cycle progression in GC-2 cells. The intercellular levels of hsa-miR-196a-5p were confirmed by qPCR at 24 h after transfection. We found that the rate of apoptosis was significantly increased in GC-2 cells transfected with hsa-miR-196a-5p mimics compared to control cells, whereas decreased in cells transfected with inhibitors at 24 h (Fig. 2). However, no significant difference was observed in cell proliferation and cell cycle progression (Fig. 2).

### Enrichment analysis of target genes of hsa-miR-196a-5p

Enrichment analysis was performed using the GO enrichment analysis and visualization web-based bioinformatics tool GOrilla (http://cbl-gorilla.cs.technion.ac.il)^26^. A total of 10016 target genes were entered into the enrichment analyses of which 9842 were recognized by the software. 5350 duplicate genes were removed (keeping the highest ranking instance of each gene) leaving a total of 4492 genes. Only 4370 of these genes are associated with a GO term. A total of 8 enriched biological processes (Additional file 4, Additional file 5) were identified (P-value < 1 × 10^−3^). Among the most enriched processes, we found ‘positive regulation of calcineurin-NFAT signaling cascade’ with an enrichment of 85.59, but we also find other processes among the most enriched processes including ‘regulation of release of sequestered calcium ion into cytosol by sarcoplasmic reticulum’, ‘wybutosine metabolic process’ and ‘wybutosine biosynthetic process’. Other processes of interest among the most enriched are ‘macromolecule metabolic process’, ‘type II pneumocyte differentiation’, ‘cellular macromolecule biosynthetic process’ and ‘regulation of ryanodine-sensitive calcium-release channel activity’.

## Discussion

Although it has been identified that alteration of miRNAs caused by SNPs plays a crucial role in various diseases, there has been no study concerning the potential role of SNPs in miRNA genes in idiopathic male infertility. In this case-control study, for the first time, we analyzed the association between three SNPs (rs11614913, rs2910164 and rs3746444) located at the pre-miRNA regions of *hsa-mir-196a-2*, *hsa-mir-146a* and *hsa-mir-499* and the risk of idiopathic male infertility. We found that rs2910164 and rs3746444 were not associated with idiopathic male infertility, but rs11614913 in *hsa-mir-196a-2* was significantly associated with idiopathic male infertility in this Han-Chinese population. This CC genotype held about a 1.53-fold increased risk of idiopathic male infertility when compared to TT genotype. Consequently, the rs11614913 C allele may be risk factors for idiopathic male infertility in Han-Chinese population. In order to figure out which semen parameter was affected, we further divided the case group with abnormal semen parameters on the basis of sperm concentration and sperm motility: normozoospermia, asthenospermia, oligozoospermia and azoospermia. In the stratified analysis of this study, we found that rs2910164 and rs3746444 were not associated among different subgroups, while rs11614913 was associated with asthenospermia, oligozoospermia and azoospermia. Therefore, men harboring the rs11614913 C allele would have an increased risk of male infertility associated with poor sperm concentration and sperm motility, and our data confirmed that the variant of *hsa-mir-196a-2* rs11614913 was related to male infertility with abnormal semen parameters. These results suggested that *hsa-mir-196a-2* rs11614913 has a relationship with sperm concentration and sperm motility.

Recently, miRNAs have been increasingly implicated in the control of various biological processes, including cell differentiation, cell proliferation, development and apoptosis, and many pathological processes such as cancer^27^, and cardiovascular disease^28^. MiRNA profiling studies have revealed that many miRNAs are up- or down regulated in idiopathic male infertility and that most of them are up regulated^29^. MiRNAs act as negative regulators of gene expression level by inhibiting translation or promoting degradation of target mRNAs.

*R*s11614913 T > C in *hsa-mir-196a-2* and rs3746444 A > G in *hsa-mir-499* are all located in their corresponding 3p mature miRNA regions, and they may have influence on both the binding of target mRNAs to 3p mature miRNAs and pre-miRNA maturation of 5p and 3p miRNAs^30^. *HOXB8*, the target of hsa-miR-196a-5p^31^, is essential to myeloid differentiation and limb development^32^. Pre-miRNA polymorphisms can affect the mature miRNA levels, as demonstrated for *hsa-mir-196a-2* T which decrease the levels of mature hsa-miR-196a-5p. One report showed that up regulated hsa-miR-196a-5p affects mRNA expression level of the *HOX* family and AKT signaling pathway^33^,^34^, which are linked to endometriosis^34^. To date, many studies have examined the effect of the rs11614913 T > C polymorphism in *hsa-mir-196a-2* on human cancers, including lung cancer^11^, squamous cell carcinoma of head and neck^35^, but few have demonstrated this effect on idiopathic male infertility.

As mentioned above, the targets of hsa-miR-196a-5p is closely associated with cellular proliferation and differentiation. Pre-miRNA polymorphisms are likely one of many ways that the expression pattern of mature miRNAs and their targets, like cellular proliferation factors, can be influenced. Our data suggested that T > C polymorphism in *hsa-mir-196a-2* could contribute to idiopathic male infertility and should be considered when evaluating idiopathic male infertility patients. However, to date, no tangible evidence showed associations between miRNA SNPs and idiopathic male infertility, and the precise mechanisms regulating miRNA expression are ambiguous and the low penetrance genetic effect of miRNA SNPs to idiopathic male infertility diagnosis is largely unknown, but studies have suggested several mechanisms, including genetic and epigenetic alterations^36^,^37^. Because small variation in the quantity of miRNAs may have an effect on thousands of target mRNAs and result in diverse functional consequences, the most common genetic variation, SNPs, in miRNA sequences may also be functional and therefore may represent ideal candidate biomarkers for diseases diagnosis.

In this present study, expression level of hsa-miR-196a-5p in seminal plasma from idiopathic infertile males and fertile controls were measured. We found that the expression of hsa-miR-196a-5p in idiopathic infertile males were significantly higher than that in the fertile controls. Moreover, we found that the CC genotype of rs11614913 was significantly associated with increased expression level of hsa-miR-196a-5p in idiopathic infertile males. In addition, apoptosis levels of GC-2 cells were increased when overexpression of hsa-miR-196a-5p, while decreased in hsa-miR-196a-5p-inhibitor treated GC-2 cells. Altered expression patterns of hsa-miR-196a-5p can influence its targets^38^ and, therefore, might play a role in the subtle temporal and spatial regulation processes in the process of spermatogenesis and spermatozoal maturation. It supports the need for further research on the biological mechanism of the elevated hsa-miR-196a-5p expression in infertile males.

Several limitations need to be addressed. First, our study found the association between *hsa-mir-196a-2* T > C polymorphism and idiopathic male infertility in Han Chinese population, confirmations in other ethnic population are needed. Second, the sizes of oligozoospermia and azoospermia subgroups were relatively small, and may lack sufficient statistical power to explore the real association. Third, gene-gene and gene-environmental factors interactions were not addressed in this study for the lack of sufficient data. In a word, a number of further investigations regarding *hsa-mir-196a-2* T > C polymorphism and male infertility risk are required.

In conclusion, the present study demonstrates for the first time that rs11614913 in *hsa-mir-196a-2* is associated with the increased male infertility risk in Han Chinese population. *hsa-mir-196a-2* T > C polymorphism may increase the expression level of mature hsa-miR-196a-5p and consequently induce apoptosis, which could result in poor semen quality of ejaculated sperm. Larger sample size studies with different ethnic population and *in vivo* or *in vitro* functional studies are needed to substantiate the biological roles of the variant in future research.

## Methods

### Study population and sample collection

This study was approved by the ethics review board of Nanjing Medical University. All activities involving human subjects were done under full compliance with government policies and the Helsinki Declaration. The methods were carried out in accordance with the approved guidelines. From March 2005 to December 2011, 1378 males with definite idiopathic infertility and 486 fertile controls were consecutively recruited from the Affiliated Hospitals of Nanjing Medical University (NJMU Infertility Study). Controls were proved fertile healthy men who were guaranteed without any major change in their environment between the pregnancy and sample collection. Cases had been unable to conceive for at least 12 months and had undergone a complete historical and physical examination. Men with abnormal sexual and ejaculatory functions, immune infertility, infection or other agents suspected to be associated with male reproduction, were excluded from the study. Furthermore, man with Y chromosome microdeletions was also excluded^39^. All participants were ethnic Han-Chinese and provided written informed consent. After completing a questionnaire including detailed information, such as age, cigarette smoking, alcohol drinking and abstinence time (Abs), each subject donated 5 ml of blood which was used for genomic DNA extraction and an ejaculate for semen analysis.

### Semen quality analysis

Semen samples were obtained after a recommended 3-day sexual abstinence. After liquefaction at 37 °C for 30 min, semen analysis was performed by the computer-assisted semen analysis system (CASA, WLJY-9000; Weili New Century Science and Tech Dev, Beijing, China) according to World Health Organization guidelines (World Health Organization, 2010) including semen volume, sperm concentration, sperm count per ejaculate and sperm motility. Two parameters of semen quality including sperm concentration and sperm motility were chosen for the statistical analysis. Strict quality control was enforced throughout the study. Values for semen parameters were the mean of at least two analyses.

### SNP Selection and Genotype analyses

We selected three common miRNA SNPs (rs11614913, rs2910164, and rs3746444) located at the pre-miRNA regions of *hsa-mir-196a-2, hsa-mir-146a* and *hsa-mir-499*, respectively, for genotyping in this study. Our rational hypothesis was that SNPs located at pre-miRNA regions may directly affect binding of targeting mRNA and the pre-miRNA maturation process. All three SNPs were genotyped by using TaqMan SNP Genotyping Assays (Applied Biosystems, Valencia, CA, USA) following the manufacturer’s instructions. The TaqMan SNP Genotyping Assays are composed of primer and probes that are pre-designed and validated by the company (Biosteed, Nanjing, China). The probes are conjugated with HEX-MGB or FAM-MGB dyes, one to each allele. Once the PCR is complete, the 7900HT Sequencing Detection System (Applied Biosystems, Valencia, CA, USA) distributes the data points according to the signals generated depending on the allele composition of each sample. For quality control, 10% of the samples were randomly genotyped again, and the reproducibility was 100%.

### Seminal plasma collection and RNA isolation

Semen samples were first centrifuged for 5 min at 12000 rpm (13400 g). One hundred microliters of supernatant (seminal plasma) were used for total RNA isolation. Total RNA was isolated from seminal plasma using equal volume of TRIzol Reagent (Invitrogen, Carlsbad, CA, USA), according to the manufacturer’s protocol. Three steps of phenol/chloroform purification were added in order to get rid of proteins. The concentration and purity of RNA were determined by using NanoDrop^®^ ND-1000, while its quality was verified by denaturing agarose gel electrophoresis.

### Quantitative RT–PCR (qPCR)

Complementary DNA (cDNA) was synthetized from total RNA using miRNA-specific primers according to the TaqMan MicroRNA Assay protocol (Applied Biosystems, Valencia, CA, USA). qPCR analysis of miRNA expression was described previously^40^. All real-time reactions, including no-template controls and real-time minus controls, were run using the ABI 7900 Real-Time PCR System (Applied Biosystems, Valencia, CA, USA) and performed in triplicate. RNU6B (Applied Biosystems, Valencia, CA, USA) served as an endogenous control. Relative expression was calculated using the Ct values provided by the manufacturer.

### Cell culture and transfections

GC-2 cells were obtained from American Type Culture Collection (ATCC, Manassas VA, USA) maintained in high glucose DMEM with 10% fetal bovine serum (FBS), 100 U/mL penicillin and 100 μg/mL streptomycin under a humid atmosphere including 5% CO_2_ at 37 °C. For transfection, the hsa-miR-196a-5p mimics and inhibits-containing media (without the addition of antibiotics) were added when the cells reached 70% confluence. The cells were incubated with 50 nM mimics, 100 nM inhibit for 24 hours. The intercellular levels of hsa-miR-196a-5p were evaluated by qPCR at 24 h after transfection

### Cell proliferation assays

GC-2 cells were seeded in a 96-well plate at a density of 5, 000 cells/well and incubated overnight. After treatment with hsa-miR-196a-5p mimics and inhibit for 24 h, we added 10 μl CCK-8 (Beyotime, Haimen, China) to each well. Plates were incubated at 37 °C for 1 h, and then measured at 450 nm by the Tecan Infinite M200 multimode microplate reader (Tecan, Mechelen, Belgium). Each assay were performed in triplicate and repeated three times.

### Cell cycle and apoptosis analysis

GC-2 cells in the exponential growth phase were transfected for 24 hours with hsa-miR-196a-5p and then harvested by trypsinization. For the cell cycle assay, the cells were washed with PBS twice and then fixed with 75% ethanol at −20 °C and stained with propidium iodide (Sigma, MO, USA). After washing twice, we measured apoptosis by Annexin V-FITC Apoptosis Detection Kit (BD Biosciences, Franklin Lakes, NJ, USA). All experiments were analyzed by BD Biasciences FACS Calibur Flow Cytometry (BD Biosciences, Franklin Lakes, NJ, USA). The cell cycle fraction was determined using Modfit LT version 3.0 software. Each assay were performed in triplicate and repeated three times.

### Bioinformatics analysis of target genes of hsa-miR-196a-5p

The target genes of *hsa-miR-196a-5p* were predicted by miRWalk software^41^. We performed the enrichment analysis in GOrilla using a mode of single ranked list of genes. The analysis will aid to identify GO terms (molecular function and biological processes) that are significantly enriched for genes potentially regulated by hsa-miR-196a-5p.

## Statistical analyses

Pearson’s chi-squared test was used to analysis the differences of categorical variables such as smoking and alcohol status between cases and controls. Student’s t test was used to test for differences in continuous variables such as age, body mass index (BMI), abstinence time and ejaculate volume. Semen parameters were dichotomized based on WHO reference values. The subjects with idiopathic infertility were first treated as total and then divided into four subgroups according to semen parameters: normospermia (sperm concentration >=15 × 10^6^/ml and progressive motility >=32%), asthenospermia (progressive motility < 32%), oligozoospermia (sperm concentration < 15 × 10^6^/ml) and azoospermia (sperm concentration = 0 × 10^6^/ml). In the case groups, a subject may contribute data to more than one subgroup. Infertility risks were estimated as odds ratios (OR) and 95% confidence intervals (95% CI) using unconditional multivariate logistic regression adjusted for age, BMI, smoking status and alcohol drinking. To test the robustness of the results, we further performed the sensitivity analyses using re-sampling statistical methods including bootstrap, permutation, and delete-one jackknife^42^,^43^. The relative expression was calculated using the equation 2^–∆Ct^, in which ∆Ct = Ct_hsa-miR-196a-5p_ − Ct _U6_. Statistical analyses for hsa-miR-196a-5p expression levels were performed by Mann-Whitney test. All statistical analyses were carried out using Stata (Version 14, StataCorp LP, TX, USA), and *P* < 0.05 were considered to be significant.

## Additional Information

**How to cite this article**: Lu, J. *et al*. Common SNP in hsa-miR-196a-2 increases hsa-miR-196a-5p expression and predisposes to idiopathic male infertility in Chinese Han population. *Sci. Rep*. **6**, 19825; doi: 10.1038/srep19825 (2016).

## Supplementary Material

Supplementary Information

## Figures and Tables

**Table 1:** Characteristics of the control and case.

Variables	Control	Case	Normospermia^a^	Asthenospermia^b^	Oligozoospermia^c^	Azoospermia^d^
		Total				
	(n = 486)	(n = 1378)	(n = 927)	(n = 405)	(n = 131)	(n = 140)
Age (years, mean ± SD)	29.66 ± 3.11	28.86 ± 4.09*	28.68 ± 4.12*	29.21 ± 3.98	28.78 ± 3.90*	29.31 ± 4.67
BMI (kg/m2, mean ± SD)^e^	23.86 ± 2.62	23.58 ± 3.01	23.54 ± 2.84*	23.81 ± 3.43	23.54 ± 4.40	23.91 ± 2.79
Smoking status [n (%)]						
No	310 (63.79)	779 (56.53)	518 (55.88)	236 (58.27)	69 (52.67)	73 (52.14)
Yes	176 (36.21)	599 (43.47)*	409 (44.12)*	169 (41.73)	62 (47.33)*	67 (47.86)*
Alcohol intake [n (%)]						
No	436 (90.12)	1197 (86.87)	794 (85.65)	362 (89.38)	116 (88.55)	129 (92.14)
Yes	48 (9.88)	181 (13.13)	133 (14.35)*	43 (10.62)	15 (11.45)	11(7.86)
Abstinence time (days, mean ± SD)	4.47 ± 1.70	5.43 ± 3.53*	5.47 ± 3.26*	5.46 ± 3.91*	4.75 ±1.78	5.68 ± 2.91*
Ejaculate volume (ml, mean ± SD)	3.29 ± 1.13	3.00 ± 1.46*	3.26 ± 1.49	2.70 ± 1.29*	3.04 ± 1.54	2.39 ± 1.22*

^a^Subjects with normal sperm concentration and motility.

^b^Subjects with sperm motility < 32%.

^c^Subjects with sperm concentration < 15 × 10^6^/ml.

^d^Subjects with sperm concentration = 0 × 10^6^/ml.

^e^BMI: body mass index.

*P < 0.05 for T test or two-side *X*^2^ test for selected characteristics distributions between control and case groups.

**Table 2:** Associations of three pre-miRNA polymorphisms with risk of idiopathic asthenospermia, oligozoospermia and azoospermia.

	Control	Case														
	(n = 486)	Total (n = 1378)			Normospermia^a^(n = 927)			Asthenospermia^b^ (n = 405)			Oligozoospermi^c^(n = 131)			Azoospermia^d^ (n = 140)		
	n (%)	n (%)	OR (95% CI)	*P*	n (%)	OR (95% CI)	*P*	n (%)	OR (95% CI)	*P*	n (%)	OR (95% CI)	*P*	n (%)	OR (95% CI)	*P*
*hsa-mir-196a-2* rs11614913																
TT	186 (38.27)	424 (30.77)	1.00 (Ref.)		313 (33.76)	1.00 (Ref.)		102 (25.19)	1.00 (Ref.)		26 (19.85)	1.00 (Ref.)		36 (25.71)	1.00 (Ref.)	
CT	213 (43.83)	656 (47.61)	**1.34 (1.06–1.69)**	**0.014**	427 (46.06)	1.17 (0.92–1.51)	0.204	209 (51.60)	**1.78 (1.31–2.43)**	**< 0.001**	59 (45.04)	**1.93 (1.16–3.21)**	**0.011**	68 (48.57)	**1.60 (1.01–2.51)**	**0.043**
CC	87 (17.90)	298 (21.63)	**1.53 (1.13–2.06)**	**0.005**	187 (20.17)	1.31 (0.95–1.79)	0.098	94 (23.21)	**1.99 (1.36–2.91)**	**< 0.001**	46 (35.11)	**3.87 (2.23–6.72)**	**< 0.001**	36 (25.71)	**2.20 (1.29–3.76)**	**0.005**
CT + CC	300 (61.73)	954 (69.23)	**1.39 (1.12–1.73)**	**0.003**	614 (66.23)	1.21 (0.96–1.53)	0.102	303 (74.81)	**1.84 (1.37–2.46)**	**< 0.001**	105 (80.15)	**2.48 (1.55–3.97)**	**< 0.001**	104 (74.28)	**1.76 (1.15–2.70)**	**0.009**
*hsa–mir–146a* rs2910164																
CC	92 (18.93)	248 (18.00)	1.00 (Ref.)		157 (16.94)	1.00 (Ref.)		80 (19.75)	1.00 (Ref.)		28 (21.37)	1.00 (Ref.)		26 (18.57)	1.00 (Ref.)	
CG	204 (41.98)	656 (47.61)	1.19 (0.89–1.59)	0.238	443 (47.79)	1.27 (0.93–1.73)	0.129	192 (47.41)	1.09 (0.76–1.57)	0.633	61 (46.56)	0.97 (0.58–1.63)	0.917	73 (52.14)	1.26 (0.75–2.11)	0.374
GG	190 (39.09)	474 (34.40)	0.94 (0.70–1.26)	0.674	327 (35.28)	1.03 (0.75–1.41)	0.869	133 (32.84)	0.81 (0.56–1.18)	0.273	42 (32.06)	0.73 (0.42–1.26)	0.256	41 (29.29)	0.75 (0.43–1.31)	0.320
CG + GG	394 (81.07)	1130 (82.01)	1.07 (0.82–1.40)	0.627	770 (83.06)	1.15 (0.86–1.54)	0.330	325 (80.25)	0.95 (0.68–1.34)	0.790	103 (78.63)	0.85 (0.53–1.38)	0.524	114 (81.43)	1.02 (0.63–1.66)	0.943
*hsa–mir–499* rs3746444																
AA	340 (69.96)	989 (71.77)	1.00 (Ref.)		663 (71.52)	1.00 (Ref.)		296 (73.09)	1.00 (Ref.)		94 (71.76)	1.00 (Ref.)		95 (67.86)	1.00 (Ref.)	
AG	132 (27.16)	351 (25.47)	0.91 (0.72–1.16)	0.443	236 (25.46)	0.90 (0.70–1.16)	0.404	101 (24.94)	0.89 (0.66–1.21)	0.460	33 (25.19)	0.94 (0.60–1.47)	0.781	41 (29.29)	1.13 (0.74–1.72)	0.566
GG	14 (2.88)	38 (2.76)	0.93 (0.49–1.74)	0.811	28 (3.02)	0.99 (0.51–1.91)	0.967	8 (1.98)	0.66 (0.27–1.61)	0.363	4 (3.05)	1.25 (0.39–3.95)	0.708	4 (2.86)	1.00 (0.32–3.14)	0.999
AG + GG	146 (30.04)	389 (28.23)	0.91 (0.73–1.15)	0.434	264 (28.48)	0.91 (0.71–1.16)	0.429	109 (26.91)	0.87 (0.65–1.17)	0.353	37 (28.24)	0.96 (0.62–1.49)	0.868	45 (32.14)	1.12 (0.74–1.68)	0.591

OR, odds ratios; CI, confidence interval;

^a^Subjects with normal sperm concentration and motility.

^b^Subjects with sperm motility < 32%.

^c^Subjects with sperm concentration < 15 × 10^6^/ml.

^d^Subjects with sperm concentration = 0 × 10^6^/ml.

ORs were adjusted for age, BMI, smoking status and alcohol drinking.
