# Supplementary material for: Common SNP in hsa-miR-196a-2 increases hsa-miR-196a-5p expression and predisposes to idiopathic male infertility in Chinese Han population
Source: Sci Rep. 2016 Jan 25;6:19825. doi: 10.1038/srep19825 (PMC4726409; doi:10.1038/srep19825)
Supplement: Supplementary Information [file srep19825-s1.doc]

**Common SNP in hsa-miR-196a-2 increases hsa-miR-196a-5p expression and predisposes to idiopathic male infertility in** **Chinese Han population**

Jing Lu, Hao Gu, Qiuqin Tang, Wei Wu, Beilei Yuan, Dan Guo, Yongyue Wei, Hong Sun, Yankai Xia, Hongjuan Ding, Lingqing Hu, Daozhen Chen, Jiahao Sha, Xinru Wang

**Additional file 1: Table S2. Associations of three pre-miRNA polymorphisms with risk of idiopathic asthenospermia, oligozoospermia and azoospermia using bootstrap and permutation methods**.

|  | **Control** | **Case** |  |  |  |  |  |  |  |  |  |  |  |  |  |  |
| --- | --- | --- | --- | --- | --- | --- | --- | --- | --- | --- | --- | --- | --- | --- | --- | --- |
|  | **(n = 486)** | **Total**  **(n = 1378)** | |  | **Normospermia^a^**  **(n = 927)** | | | **Asthenospermia^b^**  **(n = 405)** | | | **Oligozoospermi^c^**  **(n = 131)** | | | **Azoospermia^d^**  **(n = 140)** | | |
|  | **n (%)** | **n (%)** | **OR**  **(95%CI)** | ***P*_bootstrap_**  **(*P*_perm_)** | **n (%)** | **OR (95%CI)** | ***P*_bootstrap_**  **(*P*_perm_)** | **n (%)** | **OR**  **(95%CI)** | ***P*_bootstrap_**  **(*P*_perm_)** | **n (%)** | **OR**  **(95%CI)** | ***P*_bootstrap_**  **(*P*_perm_)** | **n (%)** | **OR (95%CI)** | ***P*_bootstrap_**  **(*P*_perm_)** |
| ***hsa-mir-196a-2* rs11614913** | | | | | | | | | | | | | | | | |
| TT | 186 (38.27) | 424 (30.77) | 1.00 (Ref.) |  | 313 (33.76) | 1.00 (Ref.) |  | 102 (25.19) | 1.00 (Ref.) |  | 26 (19.85) | 1.00 (Ref.) |  | 36 (25.71) | 1.00 (Ref.) |  |
| CT | 213 (43.83) | 656 (47.61) | **1.34 (1.06-1.69)** | **0.014**  **(0.018)** | 427 (46.06) | 1.17 (0.92-1.50) | 0.197  (0.248) | 209 (51.60) | **1.78 (1.30-2.43)** | **< 0.001**  (0.1070) | 59 (45.04) | **1.93 (1.14-3.27)** | **0.014**  (0.424) | 68 (48.57) | 1.60 (0.99-2.57) | 0.054  (0.454) |
| CC | 87 (17.90) | 298 (21.63) | **1.53 (1.13-2.06)** | **0.006**  **(0.006)** | 187 (20.17) | 1.31 (0.95-1.79) | 0.097  (0.148) | 94 (23.21) | **1.99 (1.35-2.94)** | **0.001**  (0.096) | 46 (35.11) | **3.87 (2.22-6.75)** | **< 0.001**  (0.264) | 36 (25.71) | **2.20 (1.27-3.82)** | **0.005**  (0.370) |
| CT + CC | 300 (61.73) | 954 (69.23) | **1.39 (1.11-1.75)** | **0.004**  **(0.003)** | 614 (66.23) | 1.21 (0.97-1.52) | 0.095  (0.165) | 303 (74.81) | **1.84 (1.37-2.47)** | **< 0.001**  (0.062) | 105 (80.15) | **2.48 (1.52-4.05)** | **< 0.001**  (0.354) | 104 (74.28) | **1.76 (1.13-2.75)** | **0.012**  (0.384) |
| ***hsa-mir-146a* rs2910164** | | | | | | | | | | | | | | | | |
| CC | 92 (18.93) | 248 (18.00) | 1.00 (Ref.) |  | 157 (16.94) | 1.00 (Ref.) |  | 80 (19.75) | 1.00 (Ref.) |  | 28 (21.37) | 1.00 (Ref.) |  | 26 (18.57) | 1.00 (Ref.) |  |
| CG | 204 (41.98) | 656 (47.61) | 1.19 (0.88-1.60) | 0.254  (0.240) | 443 (47.79) | 1.27 (0.93-1.74) | 0.134  (0.204) | 192 (47.41) | 1.09 (0.77-1.55) | 0.625  (0.701) | 61 (46.56) | 0.97 (0.57-1.64) | 0.918  (0.961) | 73 (52.14) | 1.26 (0.73-2.19) | 0.404  (0.647) |
| GG | 190 (39.09) | 474 (34.40) | 0.94 (0.69-1.28) | 0.689  (0.667) | 327 (35.28) | 1.03 (0.74-1.42) | 0.872  (0.864) | 133 (32.84) | 0.81 (0.56-1.18) | 0.275  (0.419) | 42 (32.06) | 0.73 (0.42-1.26) | 0.266  (0.589) | 41 (29.29) | 0.75 (0.43-1.33) | 0.327  (0.645) |
| CG + GG | 394 (81.07) | 1130 (82.01) | 1.07 (0.81-1.40) | 0.631  (0.610) | 770 (83.06) | 1.15 (0.86-1.54) | 0.334  (0.365) | 325 (80.25) | 0.95 (0.68-1.34) | 0.791  (0.8345) | 103 (78.63) | 0.85 (0.53-1.39) | 0.528  (0.768) | 114 (81.43) | 1.02 (0.62-1.68) | 0.945  (0.971) |
| ***hsa-mir-499* rs3746444** | | | | | | | | | | | | | | | | |
| AA | 340 (69.96) | 989 (71.77) | 1.00 (Ref.) |  | 663 (71.52) | 1.00 (Ref.) |  | 296 (73.09) | 1.00 (Ref.) |  | 94 (71.76) | 1.00 (Ref.) |  | 95 (67.86) | 1.00 (Ref.) |  |
| AG | 132 (27.16) | 351 (25.47) | 0.91 (0.72-1.16) | 0.449  (0.465) | 236 (25.46) | 0.90 (0.70-1.15) | 0.398  (0.449) | 101 (24.94) | 0.89 (0.65-1.22) | 0.469  (0.545) | 33 (25.19) | 0.94 (0.60-1.46) | 0.778  (0.886) | 41 (29.29) | 1.13 (0.73-1.76) | 0.583  (0.764) |
| GG | 14 (2.88) | 38 (2.76) | 0.93 (0.48-1.78) | 0.817  (0.811) | 28 (3.02) | 0.99 (0.49-1.97) | 0.968  (0.965) | 8 (1.98) | 0.66 (0.25-1.75) | 0.406  (0.476) | 4 (3.05) | 1.25 (0.33-4.70) | 0.745  (0.509) | 4 (2.86) | 1.00 (0.32-3.13) | 0.999  (0.628) |
| AG + GG | 146 (30.04) | 389 (28.23) | 0.91 (0.72-1.16) | 0.449  (0.410) | 264 (28.48) | 0.91 (0.71-1.16) | 0.436  (0.467) | 109 (26.91) | 0.87 (0.64-1.18) | 0.369  (0.469) | 37 (28.24) | 0.96 (0.62-1.50) | 0.870  (0.924) | 45 (32.14) | 1.12 (0.73-1.71) | 0.605  (0.766) |

OR, odds ratios; CI, confidence interval; *P*_bootstrap_, *P* value from bootstrap analysis; *P*_perm_, *P* value from permutation test;

^a^ Subjects with normal sperm concentration and motility.

^b^ Subjects with sperm motility < 32%.

^c^ Subjects with sperm concentration < 15×10^6^/ml.

^d^ Subjects with sperm concentration = 0×10^6^/ml.

ORs were estimated by bootstrap method with adjustment for age, BMI, smoking status and alcohol drinking.

**Additional file 2: Table S3. Associations of three pre-miRNA polymorphisms with risk of idiopathic asthenospermia, oligozoospermia and azoospermia （delete-one jackknife method）**.

|  | **Control** | **Case** |  |  |  |  |  |  |  |  |  |  |  |  |  |  |
| --- | --- | --- | --- | --- | --- | --- | --- | --- | --- | --- | --- | --- | --- | --- | --- | --- |
|  | **(n = 486)** | **Total**  **(n = 1378)** | |  | **Normospermia^a^**  **(n = 927)** | | | **Asthenospermia^b^**  **(n = 405)** | | | **Oligozoospermi^c^**  **(n = 131)** | | | **Azoospermia^d^**  **(n = 140)** | | |
|  | **n (%)** | **n (%)** | **OR**  **(95%CI)** | ***P*** | **n (%)** | **OR (95%CI)** | ***P*** | **n (%)** | **OR**  **(95%CI)** | ***P*** | **n (%)** | **OR**  **(95%CI)** | ***P*** | **n (%)** | **OR (95%CI)** | ***P*** |
| ***hsa-mir-196a-2* rs11614913** | | | | | | | | | | | | | | | | |
| TT | 186 (38.27) | 424 (30.77) | 1.00 (Ref.) |  | 313 (33.76) | 1.00 (Ref.) |  | 102 (25.19) | 1.00 (Ref.) |  | 26 (19.85) | 1.00 (Ref.) |  | 36 (25.71) | 1.00 (Ref.) |  |
| CT | 213 (43.83) | 656 (47.61) | **1.34 (1.06-1.70)** | **0.015** | 427 (46.06) | 1.17 (0.91-1.51) | 0.208 | 209 (51.60) | **1.78 (1.30-2.43)** | **< 0.001** | 59 (45.04) | **1.93 (1.15-3.25)** | **0.013** | 68 (48.57) | **1.60 (1.00-2.53)** | **0.048** |
| CC | 87 (17.90) | 298 (21.63) | **1.53 (1.13-2.06)** | **0.006** | 187 (20.17) | 1.31 (0.95-1.80) | 0.099 | 94 (23.21) | **1.99 (1.36-2.92)** | **< 0.001** | 46 (35.11) | **3.87 (2.20-6.80)** | **< 0.001** | 36 (25.71) | **2.20 (1.28-3.80)** | **0.005** |
| CT + CC | 300 (61.73) | 954 (69.23) | **1.39 (1.11-1.74)** | **0.003** | 614 (66.23) | 1.21 (0.96-1.53) | 0.105 | 303 (74.81) | **1.84 (1.37-2.47)** | **< 0.001** | 105 (80.15) | **2.48 (1.53-4.03)** | **< 0.001** | 104 (74.28) | **1.76 (1.14-2.72)** | **0.010** |
| ***hsa-mir-146a* rs2910164** | | | | | | | | | | | | | | | | |
| CC | 92 (18.93) | 248 (18.00) | 1.00 (Ref.) |  | 157 (16.94) | 1.00 (Ref.) |  | 80 (19.75) | 1.00 (Ref.) |  | 28 (21.37) | 1.00 (Ref.) |  | 26 (18.57) | 1.00 (Ref.) |  |
| CG | 204 (41.98) | 656 (47.61) | 1.19 (0.89-1.59) | 0.242 | 443 (47.79) | 1.27 (0.93-1.74) | 0.132 | 192 (47.41) | 1.09 (0.76-1.57) | 0.636 | 61 (46.56) | 0.97 (0.57-1.65) | 0.918 | 73 (52.14) | 1.26 (0.75-2.14) | 0.384 |
| GG | 190 (39.09) | 474 (34.40) | 0.94 (0.70-1.26) | 0.677 | 327 (35.28) | 1.03 (0.74-1.42) | 0.871 | 133 (32.84) | 0.81 (0.56-1.18) | 0.277 | 42 (32.06) | 0.73 (0.42-1.26) | 0.266 | 41 (29.29) | 0.75 (0.43-1.33) | 0.327 |
| CG + GG | 394 (81.07) | 1130 (82.01) | 1.07 (0.82-1.40) | 0.630 | 770 (83.06) | 1.15 (0.86-1.54) | 0.335 | 325 (80.25) | 0.95 (0.68-1.34) | 0.792 | 103 (78.63) | 0.85 (0.52-1.40) | 0.532 | 114 (81.43) | 1.02 (0.62-1.67) | 0.944 |
| ***hsa-mir-499* rs3746444** | | | | | | | | | | | | | | | | |
| AA | 340 (69.96) | 989 (71.77) | 1.00 (Ref.) |  | 663 (71.52) | 1.00 (Ref.) |  | 296 (73.09) | 1.00 (Ref.) |  | 94 (71.76) | 1.00 (Ref.) |  | 95 (67.86) | 1.00 (Ref.) |  |
| AG | 132 (27.16) | 351 (25.47) | 0.91 (0.72-1.16) | 0.445 | 236 (25.46) | 0.90 (0.70-1.16) | 0.405 | 101 (24.94) | 0.89 (0.66-1.21) | 0.464 | 33 (25.19) | 0.94 (0.59-1.49) | 0.785 | 41 (29.29) | 1.13 (0.74-1.73) | 0.572 |
| GG | 14 (2.88) | 38 (2.76) | 0.93 (0.49-1.77) | 0.815 | 28 (3.02) | 0.99 (0.50-1.93) | 0.967 | 8 (1.98) | 0.66 (0.26-1.68) | 0.385 | 4 (3.05) | 1.25 (0.31-4.98) | 0.755 | 4 (2.86) | 1.00 (0.29-3.43) | 0.999 |
| AG + GG | 146 (30.04) | 389 (28.23) | 0.91 (0.73-1.15) | 0.436 | 264 (28.48) | 0.91 (0.71-1.16) | 0.429 | 109 (26.91) | 0.87 (0.65-1.17) | 0.357 | 37 (28.24) | 0.96 (0.62-1.50) | 0.870 | 45 (32.14) | 1.12 (0.74-1.69) | 0.595 |

OR, odds ratios; CI, confidence interval;

^a^ Subjects with normal sperm concentration and motility.

^b^ Subjects with sperm motility < 32%.

^c^ Subjects with sperm concentration < 15×10^6^/ml.

^d^ Subjects with sperm concentration = 0×10^6^/ml.

ORs were adjusted for age, BMI, smoking status and alcohol drinking.

ORs were obtained from jacknife method.

**Additional file 3: Figure S1. Physical map of rs11614913 in hsa-mir-196a-2 gene and mature miRNA hsa-miR-196a-5p in the genomic.** Red arrow and red frame represent the position of the polymorphism of rs11614913 and mature miRNA hsa-miR-196a-5p, respectively.


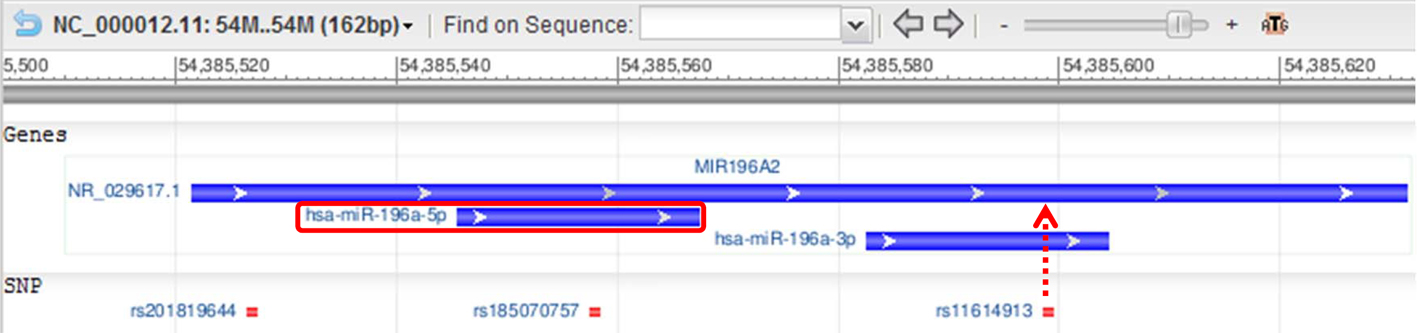


**Additional file 4: Figure S2. Biological processes that were enriched for predicted target genes of hsa-miR-196a-5p.** The white and yellow processes represent enrichment *P*-value >10^-3^ and <10^-3^, respectively.


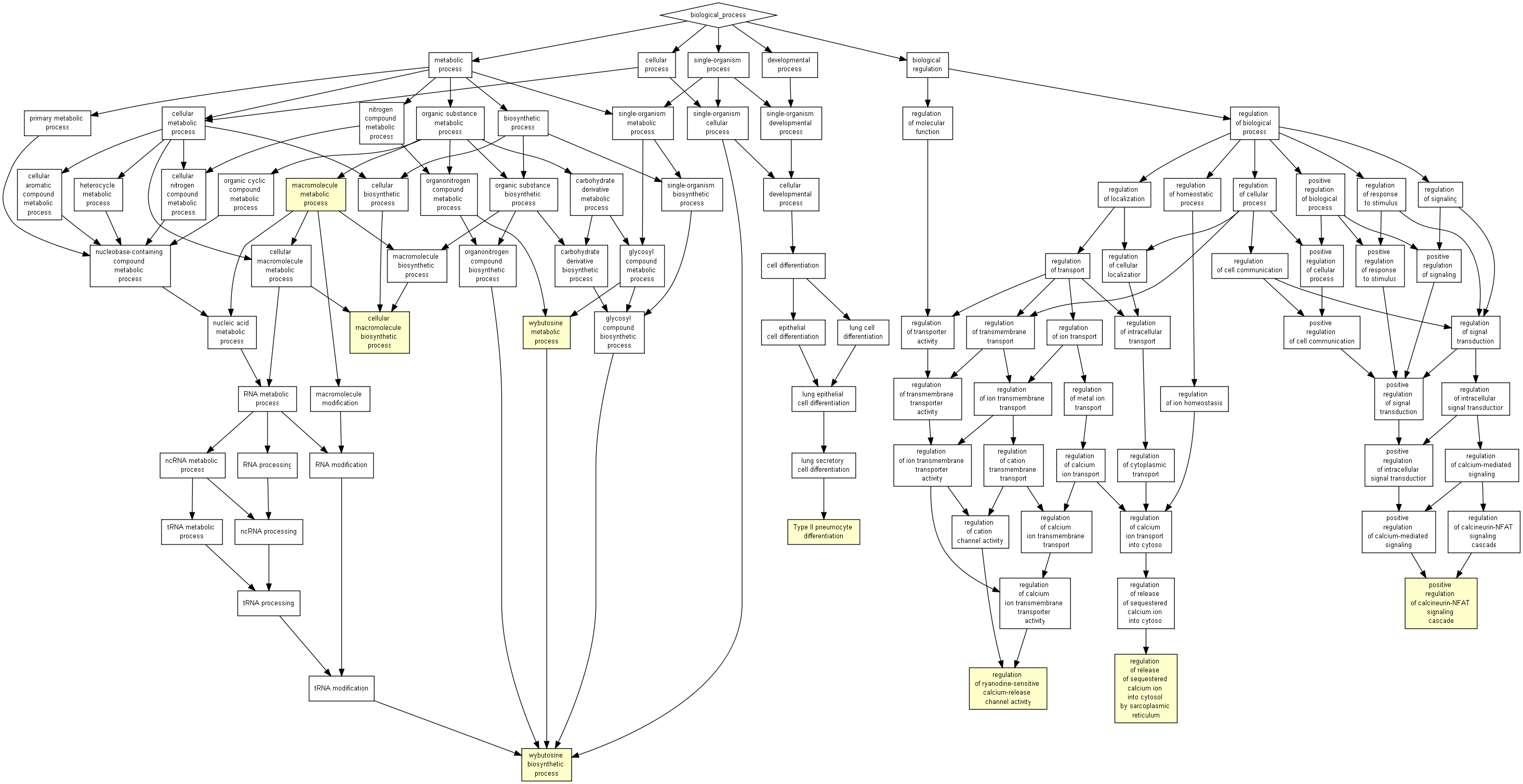


**Additional file 5: Table S1. Biological processes that were enriched for predicted target genes of hsa-miR-196a-5p.**

| **GO Term** | **Description** | **P-value** | | **FDR q-value** | **Enrichment** | | **N** | **B** | **n** | **b** |
| --- | --- | --- | --- | --- | --- | --- | --- | --- | --- | --- |
| GO:0070886 | Positive regulation of calcineurin-NFAT signaling cascade | | 1.34E-4 | 1E0 | | 85.59 | 4365 | 2 | 51 | 2 |
| [GO:0010880](http://www.godatabase.org/cgi-bin/amigo/go.cgi?query=GO:0010880&view=details) | Regulation of release of sequestered calcium ion into cytosol by sarcoplasmic reticulum | | 3.83E-4 | 1E0 | | 32.74 | 4365 | 8 | 50 | 3 |
| [GO:0031590](http://www.godatabase.org/cgi-bin/amigo/go.cgi?query=GO:0031590&view=details) | Wybutosine metabolic process | | 4.58E-4 | 1E0 | | 2.16 | 4365 | 1 | 2 | 1 |
| [GO:0031591](http://www.godatabase.org/cgi-bin/amigo/go.cgi?query=GO:0031591&view=details) | Wybutosine biosynthetic process | | 4.58E-4 | 1E0 | | 2.11 | 4365 | 1 | 2 | 1 |
| [GO:0043170](http://www.godatabase.org/cgi-bin/amigo/go.cgi?query=GO:0043170&view=details) | Macromolecule metabolic process | | 4.81E-4 | 9.46E-1 | | 2.11 | 4365 | 1862 | 669 | 334 |
| [GO:0060510](http://www.godatabase.org/cgi-bin/amigo/go.cgi?query=GO:0060510&view=details) | Type II pneumocyte differentiation | | 7.7E-4 | 1E0 | | 1.93 | 4365 | 3 | 51 | 2 |
| [GO:0034645](http://www.godatabase.org/cgi-bin/amigo/go.cgi?query=GO:0034645&view=details) | Cellular macromolecule biosynthetic process | | 7.98E-4 | 1E0 | | 1.89 | 4365 | 755 | 476 | 114 |
| [GO:0060314](http://www.godatabase.org/cgi-bin/amigo/go.cgi?query=GO:0060314&view=details) | Regulation of ryanodine-sensitive calcium-release channel activity | | 8.72E-4 | 1E0 | | 1.64 | 4365 | 10 | 50 | 3 |

‘P-value’ is the enrichment p-value (unadjusted for multiple comparisons) computed according to the mHG or HG model. ‘FDR q-value’ is the multiple-testing corrected p-value using the Benjamini and Hochberg method, whereby, for the i^th^ term (ranked according to p-value) the FDR q-value is (p-value *number of GO terms)/i. “N” is the total number of genes, “B” is the total number of genes associated with a specific GO term, “n” is the number of genes targeted by hsa-miR-196a-5p, and “b” is the number of genes in the intersection. Enrichment = (b/n) / (B/N).
